# A one-step mild acid route to fabricate high performance porous anti-reflective optical films from cationic polymeric nanolatex

**DOI:** 10.1038/s41598-020-71200-w

**Published:** 2020-08-26

**Authors:** Tong Zhang, Jiannan Jia, Yao Xiao, Binhua Shen, Zhiyong Wang, Xiaosu Yi, Xvsheng Qiao, Yan Zhao

**Affiliations:** 1 School of Materials Science and Engineering, Beihang University, Beijing, 100191 China; 2 Beijing Institute of Aeronautical Materials, Huanshan Village, No.8, Wenquan Town, Haidian District, Beijing, 100095 China; 3 State Key Laboratory of Silicon Materials & School of Materials Science and Engineering, Zhejiang University, Hangzhou, 310027 China; 4 AVIC Composites Co., Ltd, 66 Shuanghe Road, Shunyi District, Beijing, 101300 China

**Keywords:** Materials for optics, Porous materials, Solar cells

## Abstract

Porous silica anti-reflection (AR) films are of importance in solar cells’ photon harvest. However, the usual utilized method to fabricate AR films is the two-step method since the formation of porous silica NPs (first step) and silica coating sol (second step) always require chemical systems at distinct pH values. To reduce the complexity of the process, we choose cationic emulsion as an approach to produce the porosity and propose a convenient one-step route to get high-performance antireflective films. A single layer SiO_2_ anti-reflective (AR) film with high optical transmittance up to 97.5% at 740 nm was fabricated from composite sol that was made from cationic emulsion nanolatex and tetraethylorthosilicate under acid catalysis condition. After calcination, the transmittance of AR coated glasses still held the transmittance of 96% at 550 nm. Composited with SiO_2_, Al_2_O_3_, or TiO_2_ sol binders, the transmittance of AR coated glasses could be recovered as high as 97.9% at 650 nm and the pencil hardness was further strengthened up to 6H. The composite sol can keep stable at least one month at ambient temperature without any visible precipitation. Therefore, the proposed method is promising for developing high-performance AR films effectively and economically.

## Introduction

Single-layer antireflective (SLAR) coating has been widely applied to enhance optical transmission in various devices such as solar cell front substrates and optical lenses^1,2^. Generally, there are two strategies can be utilized for SLAR films to get high optical transmittances: (1) to fabricate a SLAR film with an optical thickness equal to 1/4 wavelength; (2) to prepare it with a refractive index *n*_c_ = (*n*_0_*n*_1_)^1/2^, where *n*_0_ and *n*_1_ are the refractive indices of the air and the substrate, respectively^3^. Among various methods to fabricate SLAR films, the sol–gel method is universally applied in the industry owing to its merits suitable for large area, uniform, and low-cost coatings. By sol–gel methods, the optical thickness can be easily optimized through changing coating parameters, and the refractive index can also be facilely tuned through compositing various void structures. In recent years, sol–gel methods have been intensively exploited to prepare porous SLAR films on a large scale. Mizoshita et al.^4^ reported a sol–gel SLAR film with outstanding AR performances by compositing mesoporous silica nano-spheres with hexamethyldisilazane (HDMS) alkylated surface and mesoporous silicon sol binders produced by polyethylene oxide–polypropylene oxide–polyethylene oxide (P123) block copolymer templates. Zhang et al.^5^ utilized a PAA template method to fabricate hollow silica nano-spheres under alkaline conditions and deposited it onto the glass substrate. To strengthen the mechanical performances, the final SLAR film was fabricated by filling the interspace between silica nano-spheres with acid silica sol. However, the precise methodology and evaluation for the preparation process and final coating have never be considered strictly in a real sol–gel engineering implementation^4,6^. Firstly, in practical experiments, the silica nanoparticle or hollow spheres usually coming from ammonia catalysis reaction is so hard to be applied in the sol–gel coating industry. Because they will cause the acid silica sol (as a kind of binder) tuning to a semi-solid condition and induce SLAR films with poor mechanical properties^7^. Secondly, the productivity for silica porous nanoparticles is so low and the centrifugation process is inevitable^8^. Thirdly, other template methods, such as introducing polymer molecules as templates to fabricate low refractive index coating, are extremely limited by the high silica viscosity, which leads to non-repeatability^9^.

**Figure 1:**
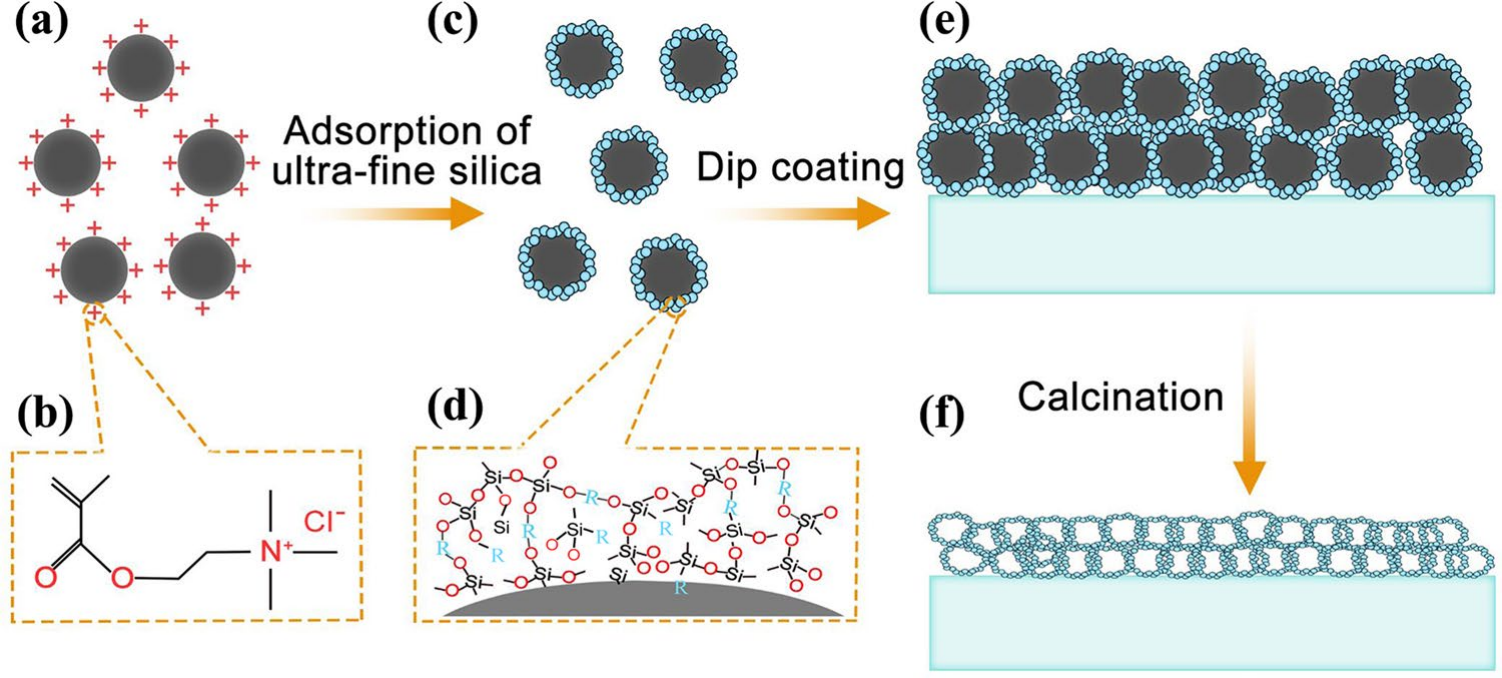
The preparation scheme of porous silica optical AR film from cationic emulsion nanolatex.

Herein, we choose cationic emulsion as an approach to adjust the porosity and propose a convenient route to get high performance antireflective. An emulsion system can be dispersed with high content suspended solids, so it has been applied as an important part of the coating industry, including both water and solvent-based coatings. A sol containing silica hollow spheres and an emulsion containing nano cationic polymer are usually chemically compatible under acid condition, so they are easy to be composited into a stabile SLAR film sol. The composite sol does not need any complex subsequent treatment (surface modification) and preforms good optical transmittance after applying to the glass substrate. The approach is illustrated in Fig. 1. Firstly, nano-scale polymer spheres are prepared. Then, by a sol–gel method, inorganic material (silica) precursor is coated on the surface of the nano-organic template to form polymer/silica core/shell spheres, which is subsequently mixed with binder sol to produce a stable coating sol. Next, the precursor coating sol is deposited on the substrate glass to prepare an uncured SLAR film. Finally, in the high-temperature tempering process, the organic matter can be removed well to achieve a porous SLAR film with enhanced mechanical properties. Since most of the emulsion is polymerized from organic monomers, it can be almost completely decomposed at high temperature (450–750 °C), cumbersome steps of removing templates in traditional preparation process are omitted. This method can be conducted easily and exhibit a huge practical application prospect in outdoor solar panels.

## Results and discussion

Previous studies^10,11^ mostly used a two-step method to prepare porous silica antireflection film. This is mainly because the formation of porous silica nano-particles (NPs) (first step) and silica coating sol (second step) require chemical systems at distinct pH values. The tetraethylorthosilicate (TEOS) is more easily hydrolyzed and polymerized under alkaline conditions to form silica NPs (first step). In contrast, as a typical binder to organize silica NPs into AR film, silica sol (binder) with chain-like internal structures is generally catalyzed and synthesized from TEOS under acidic conditions (second step), such as hydrochloric acid or acetic acid^12^. The silica prepared in the first step needs to be modified to remove the alkaline substance so that it can be uniformly dispersed in the acidic silica sol binder in the second step to form composite AR film sol. Therefore, direct exploitation of synthesizing silica NPs under acidic conditions can eliminate the cumbersome steps of removing the alkaline substance of silica NPs.

Under acidic conditions, it is inaccessible to obtain uniform silica nanospheres due to strong polycondensation. For this reason, we proposed a novel moderate method based on cationic emulsion nanolatex, as shown in Fig. 1. Because the TEOS hydrolysate is usually negatively charged, we design an oppositely charged polymer particles from organic monomers [BA (Butyl acrylate), St (Styrene) and DMC (Methacryloxyethyltrimethyl ammonium chloride)] to form cationic polymeric nanolatex (CPN) emulsion (Fig. 1a, b). After adding CPNs in acidic hydrolyzed silica sol, the negatively charged silica species will adsorb onto the CPNs surface through strong electrostatic interaction to form core–shell structures in the composite sol (Fig. 1c, d). In this strategy, DMC, the cationic monomer with ammonium, can act as a polymeric catalyst under acidic condition and/or a physical scaffold for silica deposition on the surface of CPN^13^. The hydrolysis occurs in a weak acid condition more quickly than in alkaline condition^14,15^. Subsequently, dip coating is utilized to fabricate precursor coating on substrates (Fig. 1e), which can be eventually converted into porous silica AR film after calcination (Fig. 1f). This method has the following advantages: (1) it can avoid extra steps like centrifugation or acid washing to remove ammonia^4,8,16^; (2) acid condition is beneficial to the subsequent surface modification to improve the dispersibility of composite nanoparticles^17^; (3) high solid concentration of CPNs can promise high effective productivity than other methods^18^; (4) pyrolysis of CPNs can be associated with glass manufacturing which needs high-temperature tempering process. Therefore, this method can be a promising choice to make an economical and effective AR film.

### Cationic polymeric nanolatex (CPN)

**Figure 2:**
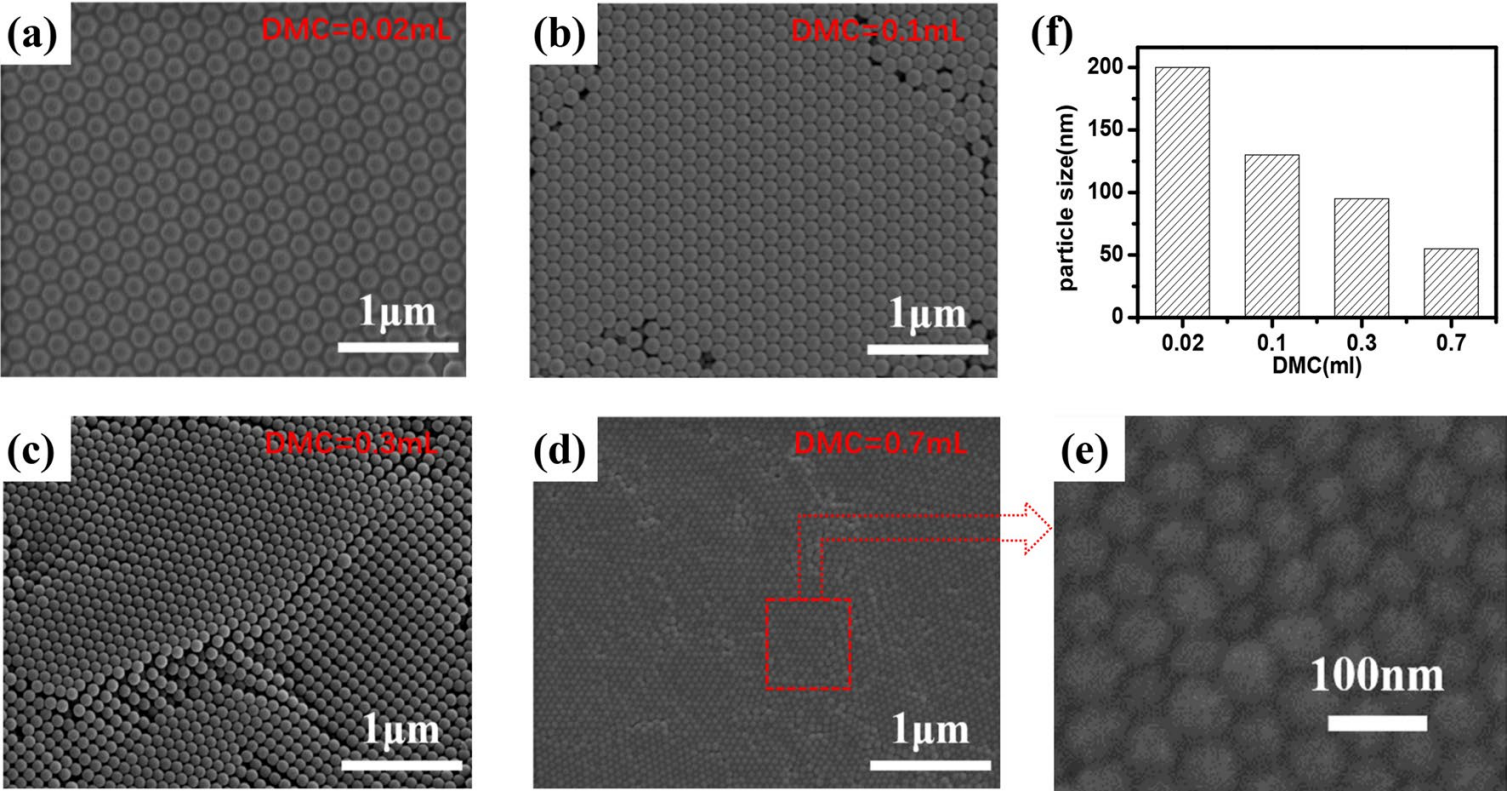
The SEM images of polymer nanolatex with different introduction of DMC: (**A**) DMC = 0.02 mL, (**B**) DMC = 0.1 mL, (**C**) DMC = 0.3 mL, (**D**) DMC = 0.7 mL.

Figure 2 clearly shows the effect of DMC on the produced CPN particle size. DMC is a highly reactive and water-soluble cationic polymerizable monomer^19^. 2,2′-azobis [2-methylpropionamidine] dihydrochloride (AIBA) acts as a cationic initiator to assist the reaction among DMC, BA, and St. Active tri-polymer segments (DMC/BA/St) are thus formed as initial nuclei to be potentially grown into larger CPN particles^20^. DMC on CPN surfaces with positive charge chemically stabilize the produced emulsion. It then avoids the sedimentation of the emulsion. With an increasing introduction of DMC, the CPN size gradually decreases. At the same time, the resulting emulsion is more stable in our experiments due to the formation of fine CPN particles with increased surface charge density. It has been previously reported^19,20^ that extremely water solubility of DMC would induce secondary nucleation at a high DMC introduction case, resulting in an uneven CPN size distribution. Those phenomena were not observed in this experiment because of the low introduction level of DMC.

**Figure 3:**
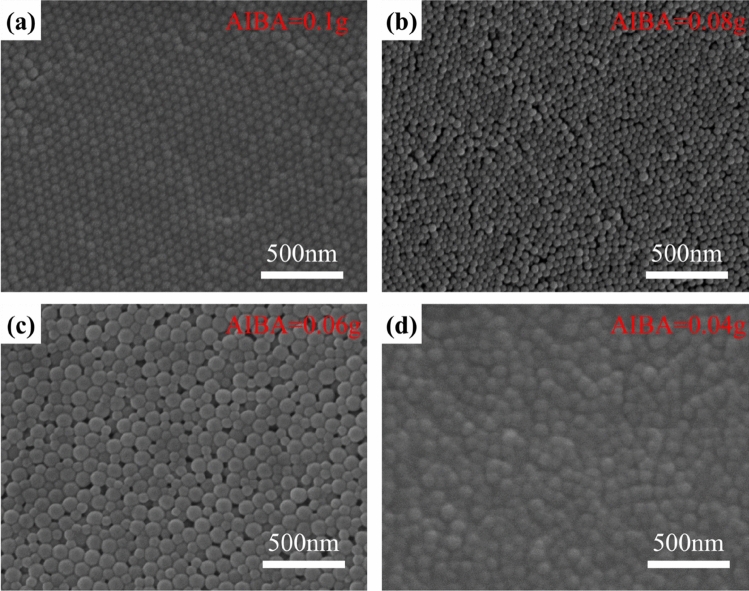
The SEM images of polymer nanolatex with different introduction of AIBA: (**a**) AIBA = 0.1 g, (**b**) AIBA = 0.08 g, (**c**) AIBA = 0.06 g, (**d**) AIBA = 0.04 g.

Figure 3 provides the evidence of the cationic initiator (AIBA) concentration effect on the CPN particle size. As the amount of AIBA decreases from 0.1 g to 0.04 g, the particle distribution changes from mono-dispersive at 55 nm to poly-dispersive at 50–100 nm. This phenomenon can be related to traditional nucleation and growth theory. A high nucleation rate leads to a small particle size of the newly formed phase, and vice versa^21^. When AIBA is reduced to a certain amount (0.06–0.04 g), the concentration of nucleation in the seed emulsion drops significantly. The subsequent monomer polymerization process can only start from a limited number of seeds, thus an individual CPN particle can grow up to a large size. A large amount of AIBA further stabilizes the produced CPN emulsion with high stability. In our experiments, those with 0.1 g or 0.08 g AIBA kept blue transparent almost without precipitation after half one year, while white precipitation emerged in those with 0.06 g and 0.04 g AIBA after one or half a month. The white precipitation is mainly from unpolymerized monomers (DMC, BA, and St) in the emulsion.

### AR film with CPN composite sol

**Figure 4:**
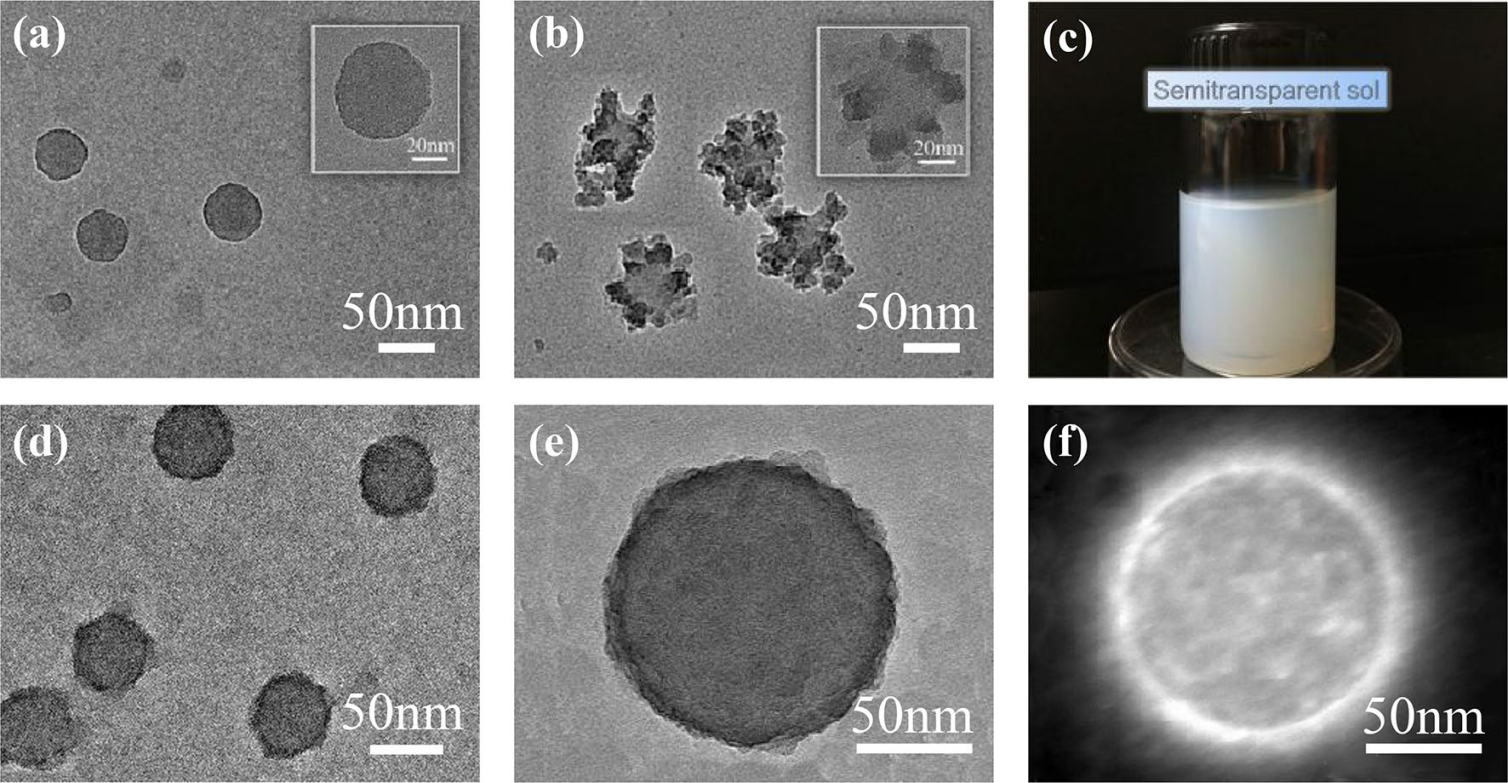
TEM (**a**, **b**, **d**, **e**) and HADDF-STEM (**f**) images of CPN/SiO_2_ core/shell nanostructures. (**c**) The photograph of CPN/SiO_2_ core/shell nanostructure composite sol.

Well-coated CPN/SiO_2_ can be obtained by controlling the core/shell precursor ratio (CPN emulsion/TEOS ratio), TEOS hydrolysis rate (acetic acid concentration, water/ethanol ratio), polycondensation rate (amino group concentration, Coulomb interaction). As shown in Fig. 4a, the uncoated CPN particles are uniformly dispersed and have a particle size of about 50 nm. Although there have been a large number of papers about polymer latex^22^, to the best of our knowledge, this is the smallest cationic nanolatex size with high uniformity via emulsifier-free emulsion polymerization. The small size of CPNs is essential to avoid scattering in the visible range. The involvement of large CPN particle and void size will result in a foggy appearance of AR film.

At an acid catalytic condition, the hydrolyzation rate of TEOS is much faster than the polycondensation rate, where polycondensation even starts before hydrolyzation of TEOS. When the ratio of CPN/TEOS is 2:1 and H_2_O: EtOH is 1: 40, the SiO_2_ partially coating on CPN (Fig. 4b) can be obtained at acid catalytic condition (pH ≈ 3). The star-like CPN/SiO_2_ nanoparticles form after the fine silica nanoparticles depositing onto the CPN surface. Although the acid sol can be stably stored for a period of time in the test, the SiO_2_ produced by hydrolysis does not completely grow on the polymer surface. According to the above mentioned reaction dynamic feature of an acid sol–gel system, for obtaining SiO_2_ well coated CPN sol, there is a lack of TEOS and H_2_O, but excessive in CPN and EtOH. Those deduce an inadequate hydrolyzation of TEOS as well as a lower production rate of SiO_2_. The dilution effect by ethanol also breaks the electrostatic repulsion balance of the CPN emulsion and induces a poor mono-dispersibility of the final product deposition^23^. It thus causes the abnormal growth of the coating layer at the local site and the unevenness of the entire surface by the polycondensation of TEOS cover. For the above reasons, the hydrolysis/polycondensation processes of an increased amount of TEOS should be controlled by adjusting the ratio of water to ethanol. Then, the ratio of CPN/TEOS is adjusted to 1:1, the ratio of water to ethanol is increased to 10:3, and the pH value is kept at 3. This can adjust the dispersibility of the latex particles in the mixed solvent and alleviate the excessive polycondensation of TEOS^24^. As a result, well coated CPN/SiO_2_ sol (Fig. 4c, d) can be eventually prepared with blue white semitransparency.

With this new route for preparing an antireflection coating sol, the silica nanoparticle obtained by the weak acid coating method does not need to be post-washed after centrifugation. The obtained nanoparticles have a uniform spherical morphology and good dispersibility, and the coating on each CPN nanoparticle is very even. To a further step, the above method is suitable to fabricate 100 nm size CPN/SiO_2_ core/shell nanoparticles (Fig. 4e, f). The contrast of the core/shell nanostructure can be recognized under the TEM (Fig. 4e) and HADDF-STEM observation (Fig. 4f). The outer surface has a bright area of about 10 nm, which is different from the core. It indicates that silica does grow on the surface of microspheres. Thus, a very stable CPN/SiO_2_ composite sol is obtained (see Fig. 4c). It can keep stable at room temperature and normal pressure for more than half a year.

**Figure 5:**
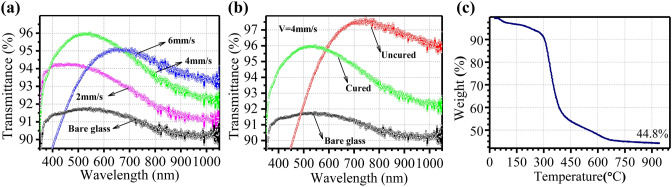
(**a**) The transmittance spectra of nanoporous silica optical AR films dip-coated with different pulling speed, (**b**) the transmittance spectra of cured and uncured nanoporous silica optical AR films dip-coated with the 4 mm/s pulling speed, (**c**) thermal gravimetric analysis (TGA) curve of dry composite sol.

The optical transmittance of the AR films (Fig. 5a) can be improved from below 92% to the maximum of 94–96% with different dip-coating parameters. For the aim to optimize the transmittance at a certain wavelength (λ), we could modify AR films as follow strategies: (1) to maximize the destructive interference by adjusting the layer thickness, *d*, to be *λ*/4*n*_c_; (2) to minimize Fresnel reflection loss by inserting a single layer AR film with an intermediate refractive index, *n*_c_ = (*n*_0_*n*_1_)^1/2^, where *n*_0_ and *n*_1_ are the refractive indices of the air and the substrate, respectively. Due to thickness directed destructive interferences and refractive index determined Fresnel reflection losses on the interface between layers, the investigated films can get good AR performances. The Λ-shape transmittance curves in Fig. 5a, b show the typical thickness-wavelength dependent destructive interference, following *d* = *λ/4n*_*c*_ equation. For single-layer ARC, it can promote the transmittance to the maximum at the wavelength “4*n*_*c*_*d*”. When the wavelength of incident light changes deviating from such specific wavelength, no complete destructive interference will happen and the transmittance would decrease rapidly. The maximum transmittance wavelength in Fig. 5a redshifts from 400 to 550 nm to 650 nm when changing the dip-coating speed from 2 mm/s to 4 mm/s to 6 mm/s and in Fig. 5b blue shifts from 750 to 550 nm before and after cured. It suggested that thickness of the single-layer ARCs increase with acceleration of the dip-coating speed and the single-layer ARCs exhibited an obvious shrinkage in thickness after cured. Those will be discussed in details with the above two strategies.

With strategy (1), we initially explored the optical behavior of AR films with various thicknesses controlled by the dip-coating speed^25^. The thickness of a dip-coating film is altered by dip-coating speed (U_0_), sol viscosity (*η*), sol density (ρ), surface tension (*r*) and the gravity constant (*g*) according to the formula (1)^14^:

1 $$ h_{0} = 0.94\frac{{\left( {{\text{U}}_{0} \eta } \right)^{{{\raise0.7ex\hbox{$2$} \!\mathord{\left/ {\vphantom {2 3}}\right.\kern-\nulldelimiterspace} \!\lower0.7ex\hbox{$3$}}}} }}{{\sqrt[6]{r}\sqrt {\rho g} }} $$

Thus, the maximum transmittance wavelength of a single layer AR film is proportional to its thickness. Therefore, the film thickness becomes large with an accelerating dip-coating. The maximum transmittance wavelength in Fig. 5a redshifts from 400 to 550 nm to 650 nm when changing the dip-coating speed from 2 mm/s to 4 mm/s to 6 mm/s.

With strategy (2), we choose the porous silica layer to reduce the reflective loss. When porous AR films are applied, the reflectance can be calculated with the formula (2)^26^:

2 $$ R = \left[ {\frac{{n_{air} n_{glass} - n_{AR}^{2} }}{{n_{air} n_{glass} + n_{AR}^{2} }}} \right]^{2} $$

Accordingly, the selection of material is a challenge because *n*_*glass*_ is 1.5151 (BK7 at 633 nm) and calculations would set *n*_*AR*_ to be approximately as low as 1.22^26^. Alternatively, we could composite a number of pores inside the films to decrease the apparent reflective index. In this case, the pores are originated from nanospheres stacking before calcination (Fig. 5a) and voids producing through removing polymer nanospheres after the calcination (Fig. 5b). The porosity as well as the apparent refractive indices are also influenced by dip-coating speed, so the absolute transmittance values of different samples in Fig. 5a are different from each other. For single-layer ARC, it can promote the transmittance to the maximum just for one single wavelength and a certain incidence angle. When the wavelength of incident light changes at a normal angle, no complete destructive interference will happen and the transmittance would decrease rapidly. Then, Λ-shape AR effect shows up in our experiment results. However, the interesting phenomenon in Fig. 5b is the maximum of optical transmittance of the AR film before calcination reaches beyond 97.5% at 740 nm, while it drops down to 96% at 550 nm after calcination. This is a consequence of overestimation of realistic refractive index. The over high porosity means the refractive index is much smaller than ideal value (1.22) resulting in less effective AR effect. Regardless of absorption and diffuse reflection, for the uncured sample, the minimum reflectance equals to 2.5% (1–0.975) occurring at the maximum transmittance peak (740 nm); for the cured sample, it equals to 4% (1–0.960) occurring at the maximum transmittance peak (550 nm). Then, according to formula (2), the apparent refractive indices could be roughly evaluated as 1.050 (uncured) and 1.006 (cured), respectively. That is to say, the refractive index of the cured sample is much farther away from the ideal refractive index (1.22) than the uncured sample. Hence, the AR effect was weakened after calcination.

The TGA curve (Fig. 5c) reveals that the coating layer lost its weight by > 50% after calcination (at 700 °C), implying increased porosity and decreased refractive index. Further assuming there is no absorption of light and according to the effective medium theory, we can calculate the porosity with the formula (3)^26^:

3 $$ P = 1 - \frac{{n_{AR}^{2} - 1}}{{n_{glass}^{2} - 1}} $$

**Figure 6:**
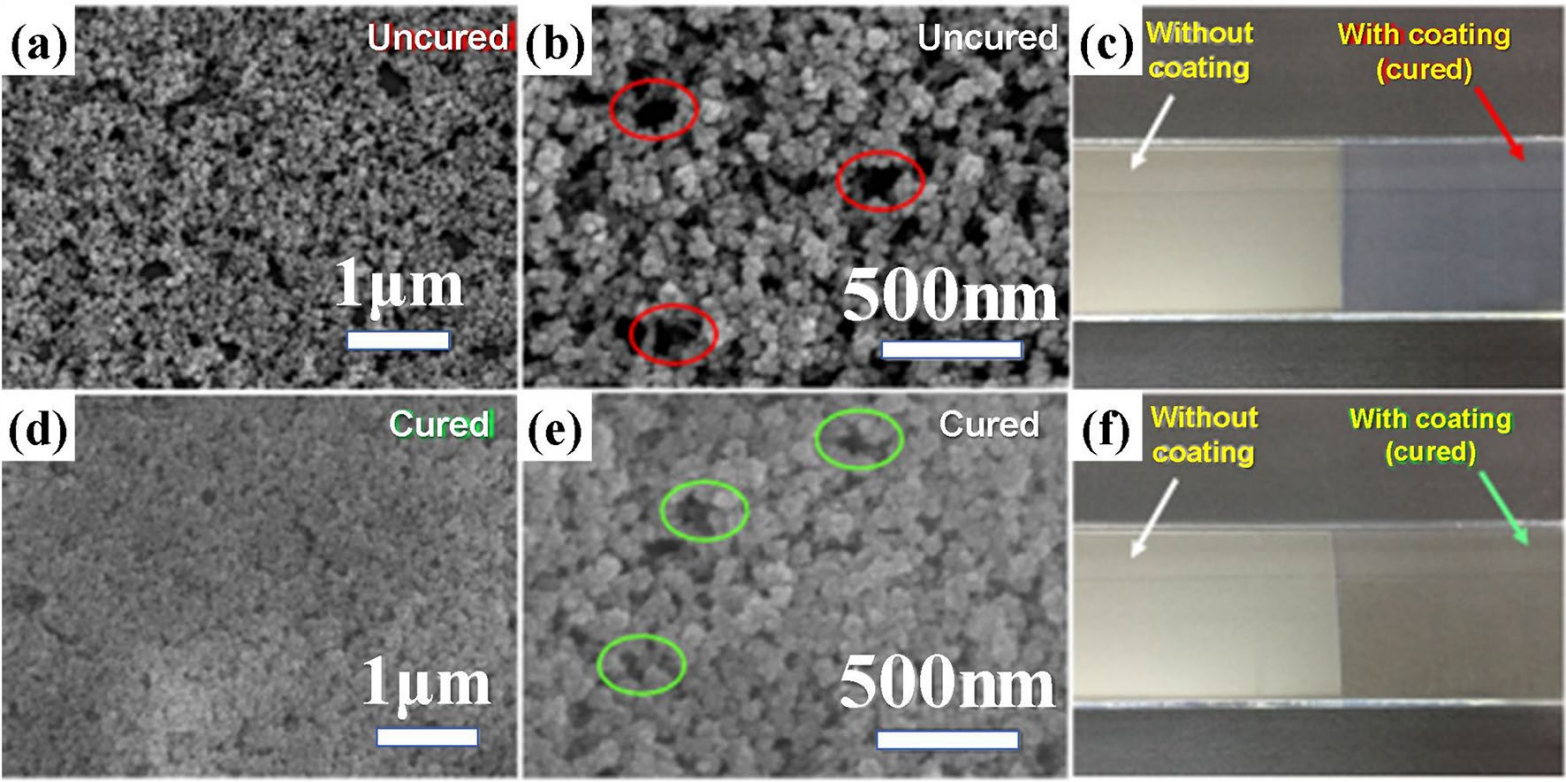
The SEM and magnified SEM images of uncured (**a,** **b**) and cured (**d,** **e**) silica optical AR films. The photographs of uncured (**c**) and cured (**f**) glasses coated with composite sol films. The circled areas in (**b,** **e**) are some loose and porous structures existed in the AR films even after calcination.

As a result, porosities are calculated as 91.08% and 99.08% for uncured and cured coatings, respectively. The estimated porosities are obviously over large, but the variation trend due to calcination is still consistent with the observed one. Figure 6 shows the SEM images of surface morphologies and photographs of the AR films before and after calcination. Both cured and uncured samples display high porosity morphologies (Fig. 6a, b, d, e) and exhibit relatively low reflectance compared to bare glass substrates (Fig. 6c, f). The uncured glass with coating exhibits a dim blue color, while the cured glass with coating appears as relatively strong red-shifted color. The reference glasses without coating both have a bright reflection. Those are consistent with the transmission spectrum in Fig. 5b.

A calcination heat treatment is usually necessary for an AR film to get strengthened in pencil hardness. Under the dip-coating speed of 4 mm/s, pencil hardness of a single layer AR film can be improved from 2H/3H before calcination to 3H/4H after calcination. However, the SEM observation (Fig. 6a, b, d, e) reveals that the surfaces of the films before and after calcination are similarly loose and porous. Those are still unfavorable to get strengthened mechanical properties for the AR films. Therefore, the overall performances of AR films should be optimized with the required thickness and porosity together. Basing on its high porosity, some typical binder sol could be introduced to enhance its mechanical properties and offset decreased refractive index.

### Strengthening with SiO_2_/Al_2_O_3_/TiO_2_ binder

Although the above AR films from the composite sol have good optical anti-reflection performances, their strengths are not yet satisfactory since there are no strong bonds between the particles. The overall mechanical properties of the film are still as low as 3H/4H. To explore the optical properties of commonly used thin film materials, we introduced some typical binder materials, such as acidic silica sol, alumina sol, and titanium oxide sol to improve the mechanical performance of porous AR films.

#### Strengthened by SiO_2_ binder

**Figure 7:**
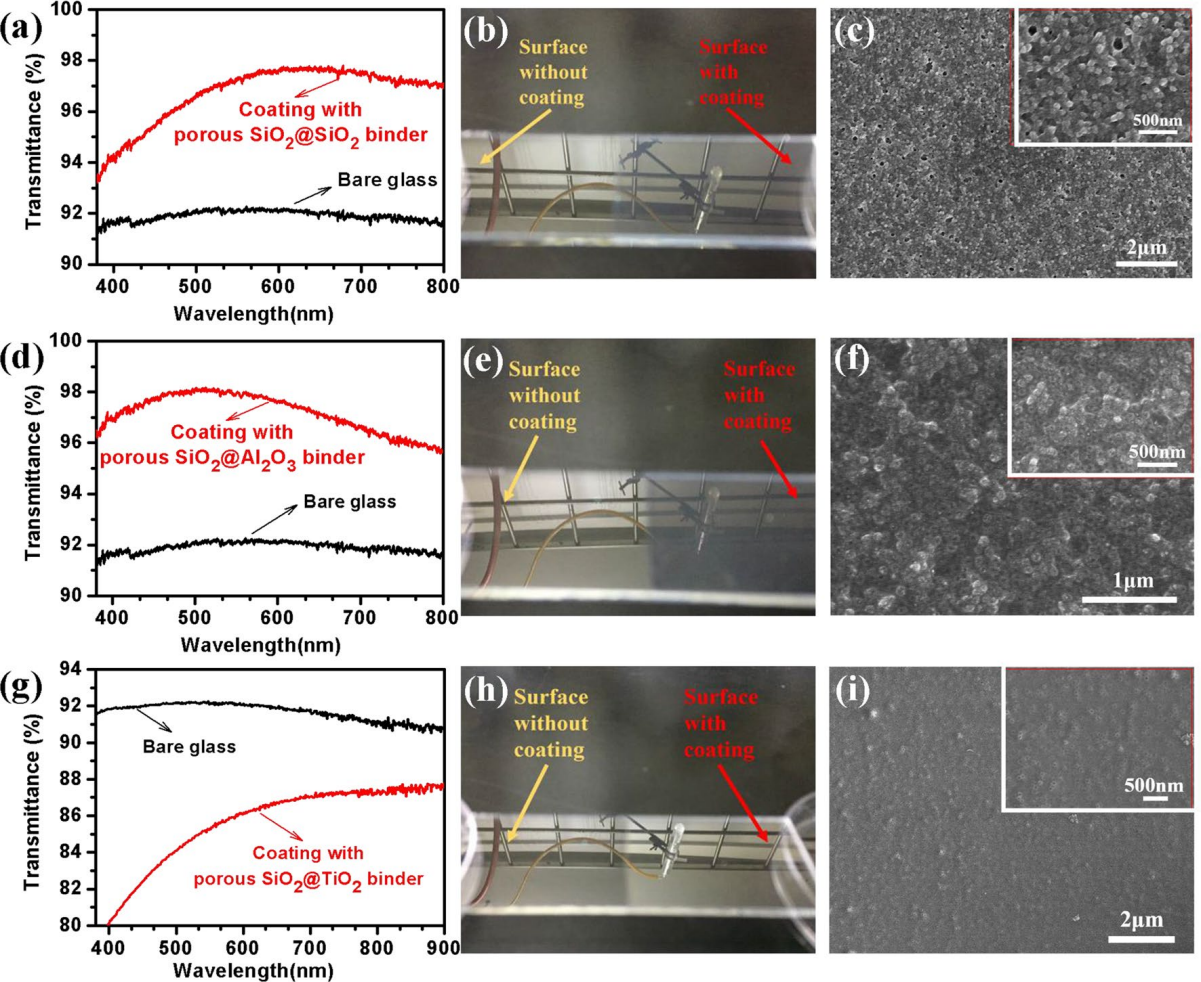
UV–VIS–NIR transmission spectra (**a**, **d**, **g**) of films using SiO_2_, Al_2_O_3_ and TiO_2_ sol as binders; photographs (**b**, **e,** **h**) and surface images (**c,** **f,** **i**) of the coated glasses.

Using SiO_2_ sol as a binder (Fig. 7a–c) can strengthen the hardness of AR films to 5H/6H and maintain maximum transmittance at 97.9%. The CPN/SiO_2_ composite sol can be directly mixed with acidic SiO_2_ sol to prepare the final AR film sol with SiO_2_ binders. The film with the SiO_2_ binder not only has a good anti-reflection effect throughout the visible spectral region but also possesses a relatively high pencil hardness. It indicates that there are strong adhesion strengths between the binder and the particles. Some literature^16^ reported that the introduction of SiO_2_ binders will lead to a loss of optical transmittance, but the opposite results were observed in our experiments. A suitable amount of SiO_2_ binder addition is helpful to enhance the mechanical properties of porous AR films. Observing different areas on the same glass (Fig. 7b), one can find the area with an AR film has a weaker optical reflection or a brighter mirror image than that without AR films. On the SEM image (Fig. 7c), the surface of the AR film with SiO_2_ binders also appears much denser than that without SiO_2_ binders (Fig. 6). Some holes are retained in the films after sintering. Hollow SiO_2_ particles are observed to be well adhered by SiO_2_ binders. The thickness of the SiO_2_ binder enhanced AR films are observed as about 100–200 nm in the TEM cross-section image, as shown in Supplementary Fig. S1 shows. The smooth silica films without other surface modifications are hydrophilic after thermal treatment, so the water contact angle is measured as about 30° as shown in Supplementary Figs. S1 and S2. It was favorable for the AR film to get a self-cleaning performance. A group of transmittance spectra (Supplementary Fig. S3) and a TEM image with low magnification (Supplementary Fig. S4) could further prove the uniformity of the coating. Therefore, a much compact, uniform, and hydrophilic porous SiO_2_@SiO_2_ binder skeleton is thus formed to support the performance improvement.

#### Strengthening with Al_2_O_3_ binder

Utilizing acidic Al_2_O_3_ sol as a binder (Fig. 7d–f) can strengthen the hardness of AR films to 6H with keeping the maximum transmittance of 98.0%. The acid Al_2_O_3_ sol was prepared by the method in the literature^27^, and it was mixed with CPN/SiO_2_ composite sol to prepare the final AR film sol with Al_2_O_3_ binders. Although the refractive index of Al_2_O_3_ is as high as 2, which is much larger than SiO_2_ or glass substrate, the AR performance and the mechanical properties of the porous SiO_2_@Al_2_O_3_ binder film are comparable with those of the porous SiO_2_@SiO_2_ binder film. Its wide-band anti-reflection performance is even better, maybe because the Al_2_O_3_ sol is positively charged and well compatible with the CPN/SiO_2_ composite sol. Thus, only a much dim mirror image (the left in Fig. 7e) can be observed at the porous SiO_2_@Al_2_O_3_ binder coating region. From the SEM image (Fig. 7f) that the pore distribution is uniform and the particles have good dispersibility. Those help the AR films achieve good optical AR and mechanical performance.

#### Strengthening with TiO_2_ binder

Utilizing acidic TiO_2_ sol as a binder (Fig. 7g–i) can strengthen the hardness of AR films to 6H, but the optical transmittance is weakened down to 80–90%. Similar to the acidic Al_2_O_3_ sol, an acidic TiO_2_ sol was prepared according to the literature, and it was mixed with CPN/SiO_2_ composite sol to prepare the final AR film sol with TiO_2_ binders. The refractive index is as high as 2.2, which is much larger than the refractive index of the inorganic silicate glass (1.4–1.5). It thus leads the AR films to get only a relatively low optical transmittance below 90% (Fig. 7g), especially in the ultraviolet-blue spectral region. This is because TiO_2_ itself is a semiconductor with a forbidden bandwidth of about 3 eV, which can absorb ultraviolet light. From the photograph (Fig. 7h), it can be observed that the strong reflection makes the AR film area much brighter than those without coating. The positively charged TiO_2_ pure sol also has so good compatibility with the CPN/SiO_2_ composite sol that the final CPN/SiO_2_ and TiO_2_ composite sol can keep uniformly dispersed without apparent precipitation for several months. The surface topography (Fig. 7i) of the film is much flatter than the other films (Fig. 7c, f). It suggests that the porous SiO_2_@TiO_2_ binder films can be applied as optics functional surfaces not in UV–Vis but NIR spectral region.

## Conclusion

Mono-dispersive CPN/SiO_2_ core/shell nano-spheres were expediently fabricated throughout an acidic condition with polymerization of template emulsion (DMC/BA/St) and a subsequent shell (SiO_2_) coating. The chemical stability and the size of CPN/SiO_2_ nano-spheres could be well adjusted by the introduction amount of DMC and AIBA. Single layer nanoporous anti-reflective (AR) films were thus made through the strategies of tuning thickness and producing void structures. Initial dip-coated CPN/SiO_2_ AR films had optical transmittance as high as 97.5% around 740 nm with much low pencil hardness (2H/3H). After calcination, CPNs were removed to form high porosity and low refractive index SiO_2_ AR film. It led the AR film to get the maximum optical transmittance down to 96% at 550 nm and the improved pencil hardness of 3H/4H. With SiO_2_ or Al_2_O_3_ binder, the AR films were further strengthened with improved pencil hardness up to 6H and held excellent optical transmittance as high as 97–98%. But the AR film strengthened by TiO_2_ only presented a much low optical transmittance (80–88%). The composite sols were chemically stable at ambient temperature at least for one month without any visible precipitation. The facile preparation method is beneficial to mass production in the practical industry.

## Materials and methods

Cationic polymeric nanolatex (CPN) were prepared by emulsion polymerization using 2,2′-azobis[2-methylpropionamidine] dihydrochloride (AIBA) (0.5 g) and cationic monomer methacryloxyethyltrimethyl ammonium chloride (DMC) (0.07 g), together with Butyl Acrylate (BA) (1 g), Styrene (St, C_8_H_8_) (5 g) and H_2_O (45 g). The polymerization was carried out in nitrogen at 70 °C for 2 h and the stirring speed was adjusted at 700 rpm. Firstly, the prepared CPN (5.0 g) was added dropwise into preformed silica sol (15 mL) and stirring vigorously, where the molar ratios of TEOS (Ethylsilicate): EtOH: H2O: AcOH (acetic acid) = 1:4:12:0.4. After stirring for 4 h, 30 mL EtOH was added into it and the composite sol was stored in a low temperature (5 °C). The sol was deposited on cleaned bare glasses using a double side dip-coating process and calcination in air (700 °C for 5 min) to remove the CPN. Parallelly, the CPN emulsion is mixed with a binder such as a TiO_2_ sol or an Al_2_O_3_ sol in a certain ratio, and coated on an ultra-white glass to obtain a corresponding functional film material, and subjected to relevant test and characterization.

The morphologies of nanoparticles (NPs) in the sol were observed by transmission electron microscope (TEM) images taken with a Tecnai G2 transmission electron microscope operated at an acceleration voltage of 200 kV. The surface morphology of the coatings was determined by scanning electron microscopy (SEM, Hitachi S-4800). The transmittance spectra of the AR films were measured by an UV–Vis–NIR (U-4100). Thermal gravimetric analysis (TGA) was used to evaluate the weight loss of composites, using TA Q500 thermal analyzer. The pencil scratch test was performed by using pencils (B-3084T3) with hardness ranging from 6B (the softest) to 6H (the hardest).

## Supplementary information

Supplementary file 1.
