# Supplementary material for: A one-step mild acid route to fabricate high performance porous anti-reflective optical films from cationic polymeric nanolatex
Source: Sci Rep. 2020 Aug 26;10:14224. doi: 10.1038/s41598-020-71200-w (PMC7450068; doi:10.1038/s41598-020-71200-w)
Supplement: Supplementary file 1 [file 41598_2020_71200_MOESM1_ESM.docx]

**Supporting Information**

**A one-step mild acid route to fabricate high performance porous anti-reflective optical films from cationic polymeric nanolatex**

Tong Zhang, ^1, 2^ Jiannan Jia, ^3^ Yao Xiao, ^3^ Binhua Shen, ^3^ Zhiyong Wang, ^2^ Xiaosu Yi, ^4^ Xvsheng Qiao^3, *^ and Yan Zhao^1, *^

^1^ School of Materials Science and Engineering, Beihang University, Beijing 100191, China

^2^ Beijing Institute of Aeronautical Materials, Huanshan Village, No.8, Wenquan Town, Haidian District, Beijing, 100095, China

^3^ State Key Laboratory of Silicon Materials, School of Materials Science and Engineering, Zhejiang University, Hangzhou 310027, China

^4^ AVIC Composites Co., Ltd, 66 Shuanghe Road, Shunyi District, Beijing 101300, China

* Correspondence authors: Email: qiaoxus@zju.edu.cn (X. Qiao); jennyzhaoyan@buaa.edu.cn (Y. Zhao)

**Table S1** Abbreviation list

| Abbreviation | Full name |
| --- | --- |
| AR  SLAR  CPN  NPs  TEM  SEM  TGA  HDMS  P123  TEOS  AIBA  DMC  BA  St | Anti-reflection  Single layer antireflective  Cationic polymeric nanolatex  Nano-particles  Transmission electron microscope  Scanning electron microscope  Thermal gravimetric analysis  Hexamethyldisilazane  Polyethylene oxide–polypropylene oxide–polyethylene oxide  Tetraethylorthosilicate  2,2'-azobis[2-methylpropionamidine] dihydrochloride  Methacryloxyethyltrimethyl ammonium chloride  Butyl Acrylate  Styrene (C_8_H_8_) |


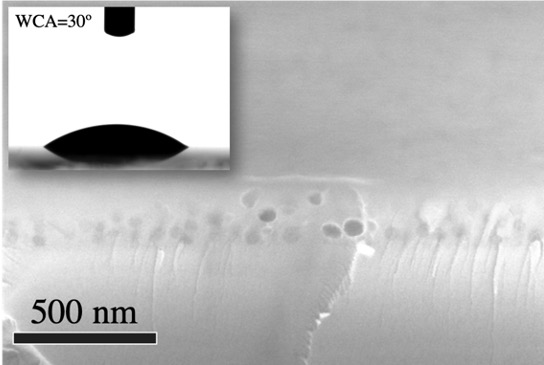


**Figure S1.** The cross-section SEM image of one SiO_2_ binder enhanced porous SLAR film. The inset shows water contact angle (WCA) on the surface of porous silica film.

**Figure S2** The water contact angle experiment results of the SiO_2_ binder enhanced porous SALR films

**Figure S3.** Transmittance spectra of four different areas at the same one AR coating with SiO_2_ sol as binder.


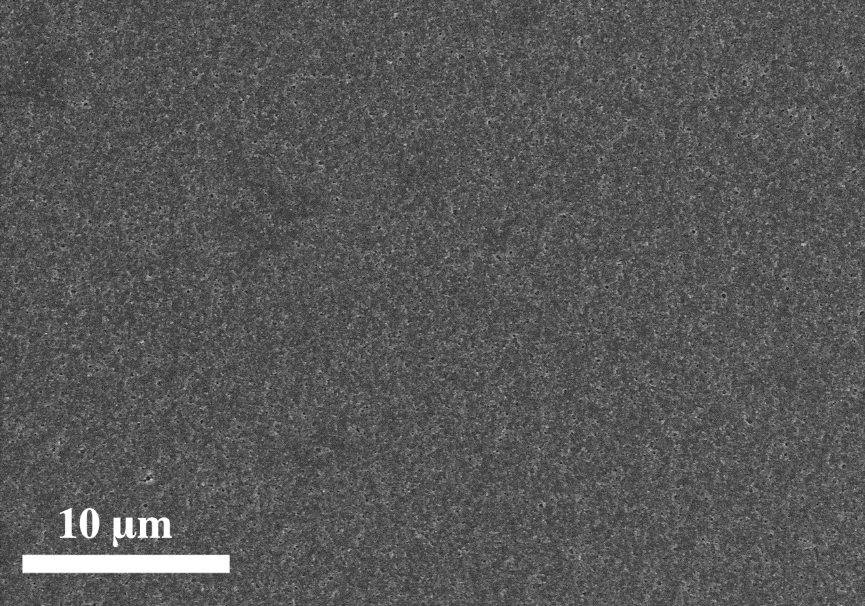


**Figure S4.** The SEM image of porous surface of AR coating using SiO_2_ sol as binder.
